# Principles of condom provision programs in prisons from the standpoint of European prison health experts: a qualitative study

**DOI:** 10.1186/s12954-021-00462-y

**Published:** 2021-01-28

**Authors:** Babak Moazen, Joy Mauti, Paula Meireles, Tereza Černíková, Florian Neuhann, Albrecht Jahn, Heino Stöver

**Affiliations:** 1 Heidelberg Institute of Global Health, Heidelberg University, Bergheimer Str. 20, Zimmer 317, 69115 Heidelberg, Germany; 2 Department of Health and Social Work, Institute of Addiction Research (ISFF), Frankfurt University of Applied Sciences, Frankfurt/Main, Germany; 3 EPIUnit—Institute of Public Health, University of Porto, Porto, Portugal; 4 Department of Addictology, First Faculty of Medicine, Charles University and General University Hospital, Prague, Czech Republic; 5 National Monitoring Centre for Drugs and Addiction, Office of the Government, Prague, Czech Republic; 6 School of Medicine and Clinical Sciences, Levy Mwanawasa Medical University, Lusaka, Zambia

**Keywords:** Condom, Sexually transmitted infections, HIV, Viral hepatitis, Prison

## Abstract

**Background:**

Condom provision is one of the most effective harm reduction interventions to control sexually transmitted infections (STIs) including HIV/AIDS and viral hepatitis in prisons. Yet, very few countries around the world provide prisoners with condoms. The present study aimed to elucidate principles of effective prison-based condom programs from the perspective of European public health and prison health experts.

**Methods:**

As a part of the “Joint Action on HIV and Co-infection Prevention and Harm Reduction (HA-REACT)” twenty-one experts from the field of prison health from eight European countries were invited to discuss the principles of condom provision programs in prisons within two focus groups. The audio records were transcribed verbatim, coded, categorized, and analyzed using thematic analysis method.

**Results:**

Six components emerged from the analysis as essential for successful condom programs in prisons: (1) highlighting the necessity of condom provision in prisons, (2) engagement of internal and external beneficiaries in all stages of designing and implementing the program, (3) conducting a pilot phase, (4) condom program in a comprehensive package of harm reduction interventions, (5) vending machine as the best method of condom distribution in prisons and (6) assuring the sustainability and quality of the intervention.

**Conclusion:**

Results of the present study can help prison health policy makers to design and conduct acceptable, accessible and high-quality prison-based condom provision programs, and consequently to mitigate the burden of STIs in prisons. Having access to high-quality healthcare services including condom provision programs is not only the right of prisoners to health, but also is a move towards achieving the sustainable development goal 3 of “leaving no one behind.”

## Background

Exceptionally high prevalence of risky behaviors including sharing contaminated injection paraphernalia, unsafe sex, as well as tattooing, piercing and the other forms of skin penetration makes prison a suitable environment for transmission of major infectious diseases (MIDs) including HIV/AIDS, viral hepatitis and tuberculosis [1]. In addition to the high-risk behaviors, a bundle of reasons including lack of access to quality healthcare services within prison amplify the vulnerability of people behind bars towards acquiring and spreading the infections [2, 3]. According to a recent estimation, globally, over 1.5 million prisoners live with hepatitis C, 491,000 with hepatitis B, 389,000 with HIV/AIDS and 286,000 with tuberculosis [4]. While prevention and treatment of MIDs are possible in today’s world, prisons can provide a unique opportunity to reach most at-risk people who are hard to access within the community. However, not all prisoners around the world have access to proper healthcare services [5].

Sexual practice, either consensual or through violence and rape, is an undeniable fact in prisons, although its practice varies and is not equally well explored in different regions of the world. It was estimated that Europe and North America (12.1%) and West and Central Africa (13.6%) had the highest level of same-sex sexual activity in prisons, while reported prevalence of this behavior was the lowest in Middle East and North African regions with 1.5% [1]. In Spain, results of a cross-sectional study found that over 34% of prisoners in Catalonia reported to have had sex while in prison [6]. Evaluation of sex and condom use among a sample of men who have sex with men in a large jail unit in the USA found a prevalence of 53% anal sex among the participants, with 75% occurring without condom [7]. Similarly, a cross-sectional multi-center health survey of sexually transmitted infections (STIs) in Mexico-City's penitentiary centers revealed that one third of male prisoners had sex in prison [8]. Since sex is a highly associated with a high taboo and denied topic in many countries, the abovementioned results are subject to underreporting.

Condom provision is one of the most effective harm reduction interventions to control STIs including HIV/AIDS and viral hepatitis in prisons [5, 9, 10]. In numerous guidelines such as the “HIV prevention, treatment and care in prisons and other closed settings: a comprehensive package of interventions” published by the United Nations Office on Drugs and Crime (UNODC), International Labour Organization (ILO), United Nations Development Programme (UNDP), World Health Organization (WHO) and Joint United Nations Programme on HIV/AIDS (UNAIDS), condom provision has been mentioned as one of the key interventions to control infection transmission in prisons [11]. Yet, only 58 countries in the world (including 28 countries in Western and Central Europe and North American region) reported distributing condom in prisons, while availability, accessibility, coverage and quality of the program in these countries are largely unknown [12].

Lack of condom provision in prisons may be due to infrastructural issues including lack of financial and human resources, or lack of explicit political will among prison policy makers. However, one of the main reasons may be misconception of prison health policy makers that condoms encourage prisoners to have sex; increase the possibility of rape and sexual assault in prisons; are used as a tool to conceal drugs and other contrabands; give the thought that most prisoners are homosexual; and give the perception that prison is a place to encourage promiscuity and homosexuality [9, 12]. On the contrary, evaluation of the long-term effects of condom provision in prisons in Australia showed that: in case of availability, condoms are more likely to be used in sex among prisoners, availability of condom did not increase sex in prisons, and condom provision leads to decreases in prevalence of STIs among prisoners [13, 14].

Considering the abovementioned phenomenon, the present study was designed and conducted to explore the characteristics of an effective condom provision program in prison from the viewpoint of European public health and prison health experts.

## Methods

On January 24, 2019, a group of public health and prison health experts including prison representatives, healthcare staff members, practitioners and researchers got together in Frankfurt to attend the conference on condom provision in prison held by the Institute of Addiction Research (ISFF), Frankfurt University of Applied Sciences. The present manuscript reports results of two focus group discussions (FGDs) held at the end of the aforementioned conference.

The conference on condom provision in prison was a part of the international research project the “Joint Action on HIV and Co-infection Prevention and Harm Reduction (HA-REACT),” funded by the Health Programme 2014–2020 from European Union (EU). Launched in late 2015, the three-year project HA-REACT aimed to find and address the gaps in prevention of the MIDs among people who inject drugs in the EU. The project was implemented by 22 research institutions representing 18 EU member states, namely Croatia, Czech Republic, Denmark, Estonia, Finland, Greece, Germany, Hungary, Iceland, Italy, Latvia, Lithuania, Luxemburg, Malta, Poland, Portugal, Slovenia and Spain. More information regarding the HA-REACT can be found on the official Web site of the project (https://www.hareact.eu/en).

The conference on condom provision in prison in Frankfurt was the final official event of the HA-REACT project. The conference was announced in December 2018 by posting a ‘call for participation’ on the online platforms (e.g., the project Web site and LinkedIn), and sending invitation letters to the research network members (including European prison health scientists, researchers, healthcare workers and policy makers) via email.

A total of 21 experts from 8 European countries including Czech Republic (2 participants), Estonia (1), Finland (1), Germany (6), Greece (1), Poland (7), Portugal (2) and the UK (1) participated in the international conference on condom provision in prison, and the focus group discussions that took place during the conference. The participants were divided into two groups to discuss the principles of condom provision programs in prisons: FGD1 consisted of 11 participants (discussing the strategies to introduce condoms in prisons) and FGD2 of 10 participants (discussing how a high-quality condom program in prison should look like).

In each group, one person was assigned to moderate the discussion and one to take notes. Both FGDs lasted around one hour and were recorded via two mobile phones. The audio records were transcribed verbatim, coded, categorized and analyzed using thematic analysis [15] method to identify characteristics of an effective condom provision program in prison. The process of coding and analysis was done by two researchers (BM and JM), manually, and discrepancies were addressed through bilateral discussions, and consultation with the project supervisor (HS).

This study has been conducted by taking ethical considerations into account. Participation in the group discussions was entirely voluntary. In the beginning, the participants were informed about aim of the discussions and our plan to publish the results in a peer-reviewed scientific journal. They were also assured that the results would be published anonymously, and their personal information would remain confidential.

## Results

The participants discussed a set of factors that need to be taken into account in designing and conducting an effective prison-based condom program. These factors have been categorized as follows: highlighting the necessity of condom provision in prisons, engagement of internal and external beneficiaries in all stages of designing and implementing the program, conducting a pilot phase, including condom program in a comprehensive package of harm reduction interventions, vending machine as the best method of condom distribution in prisons, as well as assuring the sustainability and quality of the intervention.

### Highlighting the necessity of condom provision in prisons

Often health policy makers in prisons need proof to ensure that an issue exists, and it needs to be addressed. In this case, to participants’ opinions providing evidence is necessary to convince policy makers about the existence of unprotected sex in prisons as a health challenge.

“We have some data from other countries, but from my experience, the answer [of policy makers] to that is: “But we are here in our country, and it’s not happening here. Where do you have evidence that it’s happening even here in this prison, where you want to implement something. I think that always we will hear that. That you have something evidence based, but the response to that is: “But it’s somewhere else, it’s not here. Here it is different””. (FGD1, participant 11)

“…even in a country like [name of the country redacted] with [number of the states redacted] states it is different. For example, [name of the region redacted] men are different from northern [name of the country redacted] men. So its childish, it’s ridiculous, but people would state that. They would ask for a separate study in [name of the region redacted], or elsewhere.” (FGD1, participant 3)

Instead of conducting a study to confirm that people behind bar engage in unprotected sex, highlighting the benefits of the program for people living in prisons, staff members and general population through showing a reduction in the burden of STIs would be an alternative way to convince policy makers about necessity of distributing condom among prisoners. In this regard, one of the participants stated that:

“We need no study to prove that maybe it [sex] is happening [in prisons] also in our country, because in most countries we know something about the prevalence of infectious diseases, we know how the infectious disease are being spread. We know that it is spread in prison, or at least that there is a risk of the spread in a prison. We know that those people who are in prison right now are coming back to the normal society in most cases. And there is also the prison staff who works there. I think it is better to focus on benefits for prison staff, to switch a little bit the point of view and we don’t need to make new studies if there is sex in prisons and so on. No, but just to find a way how to communicate the topic [with policy makers].” (FGD1, participant 11)

Recommended by the participants, another way to convince policy makers is to assure them that the intervention is in line with the international goals such as sustainable development goals (SDG):

“Yes, the whole operators of guidelines of sustainable development goals or that is let’s say health wise surrounding the whole discussion that we have fast track goals 2020 and so on. This also can be an argument, which wouldn’t say it is persuading anybody but it is good to have them, to argue with them. That you say: “Look, nationally we are in the position that we want to eradicate HIV 2020 by 90%,” something like that or yes, 90% of the infection, 90% are treating and 90% below have suppression of the virus. Not to argue with these overarching demands.” (FGD1, participant 3)

### Engagement of internal and external beneficiaries

According to the health experts who participated in our study, all stakeholders and beneficiaries including prisoners, prison policy makers and staff members, as well as external bodies, should be engaged in different levels of decision making to have an efficient condom provision program in prison. In this regard, one of the participants highlighted the importance of asking for prisoners’ ideas about the best place to distribute condoms in prisons as follows:

“I think it is quite useful to ask the people living in prison whether this is the best placement for them, because they know about the cameras searching them. So the best thing is to ask the prisoners find the place, the right place.” (FGD 2, participant 18)

Another FGD participant suggested to include prisoners and also prison staff and authorities:

“…and get as much factional knowledge on the table is one step, and then indeed to ask prisoners about they priorities, and ask staff with regards to healthy prison environment, what are their issues and then of course inquire also authorities and you may know from your situation, who would be rather open for that, and where would meet highest resistance.” (FGD1, participant 10)

Mentioned by an FGD participant, experience of one of the countries with prison-based condom program is an excellent example highlighting possible benefits of engaging prison authorities to guarantee the effectiveness of condom programs in prisons:

“In [name of the country redacted], they really had fears about security in prisons and we said: “OK, that’s true, that’s very important topic” and when designing the condom machines we just invited the prison authorities to help us with the design of the machine. We involved them and we let them know that security issues are very important and that we hear that. And they were so amazed, they just loved the machine and they just took it and said: “Oh yes, it’s really well done and for security in prison. Yes, yes, security on a first place”. And that helped.” (FGD 2, participant 18)

External parties including governmental, nongovernmental and international organizations could help the program through various ways including technical and financial supports, as suggested by the experts:

“Regarding the interrelationships, I think it shouldn’t be only limited to NGOs. We need to expand our vision. We need to think about all possible sources of support for our program. We should also consider receiving for example financial help from external or international organizations. We can think about for example having Ministry of Health on board, if it’s not yet involved in the program.” (FGD2, participant 5)

Engagement of social media:

“…in terms of publicity of these ideas to think about how to prepare press releases or media articles, think about how to promote it, hum, to get this social support not to, not to get the wave of reactions, hysterical reaction you give for free condoms to, to prisoners they are not doing anything, making sex.” (FGD 2, participant 18)

The participants believe that engagement can be done through building working groups consisting of different beneficiaries:

“You can even make a formal working group. In formal working group you can invite one two three more whenever you discuss the issue when you need somebody who is on top of the problem or the guards or the nurse or whatever. But then, you can also when you discuss this with management formal working group of several people who will think about, like a think tank in order to make this strategy working and convene the activity. So there should be establishing working groups.” (FGD 1, participant 6)

### To conduct a pilot phase

The next topic discussed was selection of a prison to conduct a pilot study. Conducting a pilot study would reveal the possible weaknesses and strengths of a condom program, and possibility to bridge the gaps in the post-pilot phase. According to the participants, it can also help modify the current strategy according to the needs of prisoners in a certain setting:

“Pilot takes the fears away. So people can think that they can stop it once it is not running good, they simply can stop it. Pilot is very important word then. And you should organize monitoring and evaluation. You have one paid from the project. You got paid.” (FGD 1, participant 3)

However, the participants emphasized the importance of selecting the pilot prison in a way that would avoid any stigmatization. It was important that the prison selected is not flagged out as the one that has a high level of same-sex activities in the target country:

“That’s a very sensitive topic for them [prisoners] especially when you choose one pilot prison or one pilot unit; which is usually like that you don’t implement it to all prisons in a country at the same time. And you need to communicate to them that this is not being seen as homosexual prison or homosexual unit, because that is something they are very afraid of. And it’s very important.” (FGD 1, participant 11)

As reported by the participants, prevalence of sexual activities in prisons may be attributable to the length of stay in prison. In other words, people living in prisons with long-term sentences are more likely to engage in same-sex sexual activities, compared to those who stay in prison for a short time. This phenomenon should be taken into account while choosing a pilot prison for condom programs:

“If you are talking about prisoners who are staying for a longer time, you would expect that the sexual activities would be higher. If you expect from the prisoners who are staying just a shorter period of time they would be afraid of all this idea because they would be afraid of the fact that maybe there is some sexual activities in there. Everything should be analyzed before transmitting messages and it should be considered into the strategy.” (FGD 1, participant 6)

### Condom program in a comprehensive package of harm reduction interventions

The participants believe that condom provision alone is not enough to control infection transmission in prison. To mitigate the burden of sexually transmitted infections in prisons, condom programs should be offered together with other services within a comprehensive package of harm reduction interventions. Education is one of the most important interventions that needs to be integrated with condom provision in prison:

“…you know it is not only about how to get a condom from a machine. Is about how to use that, how to dispose that. I think the term ‘sex education’ makes sense here.” (FGD 2, participant 5)

In this regard, the following quote from one of the participants from a country with condom program in prison shows how leaflets as educational material can cover various aspects of condom provision programs in prisons:

“We had two different leaflets, one was just information about the pilot project with the months of the start of the pilot project and the second leaflet was information about infectious diseases in prisons and how to protect.” (FGD 1, participant 11)

Despite the importance of education as a main intervention against infection transmission in prisons, the aim of education should not be limited to knowledge and awareness, since knowledge per se may be insufficient to make a change in prisoners’ behavior:

“I do agree that knowledge is really important, but knowledge dissemination is not necessarily the only thing that may make a change in behavior of prisoners. Most health behavior models believe that what influences the behavior of human being is not necessarily knowledge. It is attitude of people. Our program should focus on changing the attitude of prisoners.” (FGD 2, participant 5)

To enhance the effectiveness of a condom program, the package of harm reduction intervention should go beyond condom distribution and educational activities. The participants believe that condoms should be distributed together with lubricants and safe bags for disposal of the used condoms:

“…and then you should think about the package. It’s not only condoms; its lubricants and also disposable bags so everything should go in total taking care of the steps.” (FGD 1, participant 6)

### Vending machine as the best method of condom distribution in prisons

As discussed by the participants, there are various methods for distribution of condom in prisons; however, due to the stigma associated with homosexuality, prisoners may hesitate to ask for condoms. This may jeopardize the continuity of the program. In other words, condoms should be distributed anonymously with least possible personal contacts with prison staff members. The following example from a country in which condoms are available in prison canteens shows how and why prisoners hesitate to buy condoms:

“In my country prisoners have no cash. They have money on their bank accounts. They can go to the canteen and buy condoms; however, afterwards the prison system may realize that the money is dropped to buy condom; then everyone would know that this man has bought condom.” (FGD 2, participant 19)

Almost all participants believed that condom vending machines are the most appropriate method of distribution of condom among prisoners. Through condom vending machines, access to condoms will be fast and easy. Reducing personal contact, condom vending machines will also minimize the risk hesitation to access condoms due to the stigma. However, the location to install the automats should be chosen very carefully, since any mistake will jeopardize effectiveness of the program. In this regard one of the participants gave the example of a similar prison-based intervention (needle and syringe program) that failed due to choosing a wrong place to install the machines:

“It doesn’t make sense to install the automats in front of the office of the staff members. They did so in [name of the city redacted]. In [name of the city redacted] they installed the needle exchange automat in front of the window of the staff, so nobody took any syringes. Then the authorities said: “The program is not necessary. Nobody takes that.” (FGD 1, participant 3)

To address this issue, toilets and bathrooms are appropriate places in prisons to install condom vending machines. Prisoners are aware of this fact that they are not monitored through security cameras in toilets and bathrooms. As the experts recommended, that will enhance the acceptability of the program by prisoners:

“I think machine is a very convenient way. I can imagine the machines could be installed in bathroom, where prisoners could go and get condom without being watched by the security cameras.” (FGD 2, participant 18)

### Assuring the sustainability and quality

The participants mentioned that the quality of activities in each stage of implementing condom programs in prisons should be guaranteed. It is obvious that poor quality and lack of routine monitoring and evaluation will jeopardize accessibility, acceptability, sustainability and consequently effectiveness of the program.

“Quality is very important, and is not only the matter of installing a couple of condom machines and show that we have condom program. It is very important to care about the continuity and quality of the project. We need to check whether the condom machines work. We may find condom machines broken, out of service, empty or many other things. So there must be a maintenance system to make sure about that. We need to think about having a long term program.” (FGD 2, participant 5)

Suggested by the FGD participants, a routine monitoring and evaluation system will reveal possible shortcomings of each ongoing project and provide program designers with the opportunity to address the issues. Given the fact that problematic interventions will lose their clients, lack of a monitoring and evaluation system would lead to termination of the program by prison authorities. Trend of incidence of STIs, as well as satisfaction of both clients and prison staff members, is among the other factors that can be monitored and evaluated on a regular basis.

## Discussion

From the European prison health experts’ point of view, a set of components were found to be the principles of an effective prison-based condom provision program. These components can assist prison health policy makers design and implement successful prison-based condom programs, or to improve quality of the ongoing programs, and consequently mitigate the burden of STIs in prisons and communities.

There are several methods of condom distribution in prisons. In Austria, for example, at the time of admission to the facility prisoners receive a so-called health package consisting of a toothbrush, toothpaste, condom, water-based lubricant and a multilingual manual of condom use and infection transmission information [16]. Although knowledge dissemination about the necessity of condom use to control infection transmission distinguishes Austria from the other countries with condom program in prison, it seems that prisoners in Austria need to contact prison staff members to access additional condoms. This issue may jeopardize effectiveness of the program, since prisoners may hesitate to ask for condoms due to the fear of being recognized as homosexual.

In Germany, for example, condom provision in prison is very heterogeneous depending on regional and local prison health policies and practices. In all states, condoms can be acquired from the merchandiser on a one-or two-weekly basis. In some states ("Länder"), condoms are given out for free in the doctor’s office, at the social worker, chaplain/pastor. In the state of Bavaria, condoms are available on demand at the doctor’s office. This method of condom distribution is seen as ineffective, since many prisoners hesitate to ask for condoms due to fear of stigma. This underlines the necessity to design programs that bear in mind stigma and social exclusion. Condom vending machines seem to offer the best chance to provide an anonymous and confidential access to condoms and lubricants [12, 17].

In Czech Republic, condoms are being sold as an obligatory part of the range of goods in canteens in all prisons since 2007. In line with the Czech Penitentiary Concept by 2025 and its Action Plans for 2016 and 2017, free condoms were available in conjunctional visit rooms in all prisons as well. Recently, one of the most recent prison-based condom distribution programs via machines worldwide was implemented in Czech Republic and started within HA-REACT Joint Action. In August 2017, a 12-month pilot program of condom provision in one Czech prison was launched through installation of four condom vending machines that are placed in the bathrooms and toilets serving a total of 240 prisoners. Condoms are free of charge and for inmates in the rest of pilot prison units are accessible on personal request at educational staff in the prison. The pilot study showed no major problems during the implementation, and since April 2019 the condom distribution via machines was implemented in second Czech prison [12, 18].

In some countries, condoms are available only in conjugal visit rooms, rather than for sex among male prisoners [12]. Evidence shows that sexual partners of male prisoners are at high risk of acquiring major infectious diseases since they may have multiple sex partners or engage in sex work to earn money or access drugs [3]. Therefore, distribution of condoms in conjugal visit rooms is for sure an effective measure to protect infection transmissions. However, since unprotected same-sex activities occur among male prisoners, a comprehensive condom provision program in prison must cover both prisoners who have sex through conjugal visits and those who have sex with other counterparts within prisons. Ignoring one of these two groups would jeopardize infection control as the ultimate goal of condom provision programs in prisons.

In many countries with condom program in prisons, coverage of the program is either low or unknown [19]. In other words, it is unclear what proportion of prisoners has access to condoms. In the USA, for example, despite the positive results of the pilot program in San Francisco in 2007 [20] still only few states distribute condom among prisoners [12], while distributed condoms in some prisons seem insufficient [21]. Similarly, coverage of the condom provision program in prisons depends totally on the local policies [12]. Condoms must be unconditionally available and accessible to all prisoners who need them. National policies are required to oblige policy makers to initiate or expand coverage of condom provision programs in all prisons.

Introducing condom provision programs in prisons requires a systematic approach taking all the identified components into consideration. It should be taken into account that standalone interventions will fail to yield favorable results. In other words, a comprehensive package of interventions including condom, lubricant, education and risk communication, as well as screening and treatment of STIs is required to mitigate the burden of STIs in prisons. Besides, the quality of the program should go beyond the services and cover the materials (e.g., condom vending machines, condoms, and lubricants) as well. A prison-based condom provision program ignoring the abovementioned components will be incomplete.

Although the present study revealed the principles of one of the most effective interventions to control MIDs transmission in prisons, it should be seen in light of some limitations. Participants of this study were knowledgeable and experienced prison health and public health professionals; however, they all came from Europe as one of the most economically developed, and politically stable regions in the world. Another major limitation of this study was to discuss the topic with only health experts rather than prisoners and prison authorities. A more diverse group of participants from all over the world could have revealed some further aspects of prison-based condom programs during the focus group discussions. The higher number of participants from Germany and Poland than the other European countries was another limitation of the study, which may have affected the discussions and the results, and should be considered as another limitation in this study.

## Conclusions

The present study reveals principles of effective prison-based condom programs from the viewpoint of European health experts. In order to mitigate the burden of STIs in prisons, people behind bars must have access to high-quality condom provision programs in prisons. Since over 95% of prisoners will eventually return to the community, benefits of such interventions will go beyond prison walls. Having access to high-quality healthcare services including condom provision programs is not only the right of prisoners to health, but also is a move towards achieving the sustainable development goal 3 (SDG3) of “leaving no one behind.”

## Data Availability

The voice records and transcripts are available to be sent to the journal’s editors on their request.
